# KineWheel–DeepLabCut Automated Paw Annotation Using Alternating Stroboscopic UV and White Light Illumination

**DOI:** 10.1523/ENEURO.0304-23.2024

**Published:** 2024-08-23

**Authors:** Björn Albrecht, Alexej Schatz, Katja Frei, York Winter

**Affiliations:** Humboldt Universität, Berlin 10117, Germany

## Abstract

Uncovering the relationships between neural circuits, behavior, and neural dysfunction may require rodent pose tracking. While open-source toolkits such as DeepLabCut have revolutionized markerless pose estimation using deep neural networks, the training process still requires human intervention for annotating key points of interest in video data. To further reduce human labor for neural network training, we developed a method that automatically generates annotated image datasets of rodent paw placement in a laboratory setting. It uses invisible but fluorescent markers that become temporarily visible under UV light. Through stroboscopic alternating illumination, adjacent video frames taken at 720 Hz are either UV or white light illuminated. After color filtering the UV-exposed video frames, the UV markings are identified and the paw locations are deterministically mapped. This paw information is then transferred to automatically annotate paw positions in the next white light-exposed frame that is later used for training the neural network. We demonstrate the effectiveness of our method using a KineWheel–DeepLabCut setup for the markerless tracking of the four paws of a harness-fixed mouse running on top of the transparent wheel with mirror. Our automated approach, made available open-source, achieves high-quality position annotations and significantly reduces the need for human involvement in the neural network training process, paving the way for more efficient and streamlined rodent pose tracking in neuroscience research.

## Significance Statement

Animal pose tracking can help to understand the relationships between neural circuits, behavior, and neurological disorders. Training of a deep neural network requires key point annotation within the video data. To reduce human involvement in image annotation, we developed an automatic key point extraction approach for the KineWheel system.

## Introduction

Accurate, automated, and efficient methods for rodent pose tracking are essential in neuroscience research and for understanding the complex relationships between neural circuits, behavior, and neurological dysfunction (Jennings et al., 2015; Wiltschko et al., 2015; Mathis et al., 2018; Pereira et al., 2019). Traditional approaches often require time-consuming manual annotations also prone to human bias (Dollár et al., 2005; Burgos-Artizzu et al., 2012; Datta et al., 2019; Mathis et al., 2020). Automated methods for pose estimation using deep learning can track rodent behavior with minimal training data and effort and at a level of accuracy comparable to human performance (Mathis et al., 2018; Pereira et al., 2019) but at much higher speed.

These automated methods have broad applicability in neuroscience, providing insights into how animals move and interact, which in turn can be linked to the function and organization of neural circuits (Wiltschko et al., 2015; Mathis et al., 2018). Furthermore, the high-throughput collection and analysis of behavioral data facilitated by these methods have the potential to drive new discoveries in neuroscience, including the understanding of neural dysfunction and the development of treatments for neurological disorders (Pereira et al., 2019). By implementing accurate, automated, and efficient rodent pose tracking methods, researchers can gain a more detailed and precise understanding of the relationship between animal behavior and the underlying neural mechanisms (Wiltschko et al., 2015).

Pose tracking methods can be broadly categorized as marker-based or markerless (Colyer et al., 2018). Marker-based methods rely on attaching markers, such as passive reflective tape or active miniature lights, to the subject (Bernstein, 1967). In contrast, markerless methods aim to track the subject's pose directly from the video data, without the need for physical markers (Mathis et al., 2020).

Fluorescent markers are a classic marker-based tracking approach in biological research. The first synthetic fluorescent dye, fluorescein, was developed by German chemist Adolf von Baeyer in 1871 (Baeyer, 1871). The use of fluorescent labeling in biology dates far back, as seen in Pal’s (1947) study on marking mosquitoes with fluorescent compounds (Pal, 1947). Since then, fluorescent markers have been used to track animals such as insects (Stern and Mueller, 1968; Hagler and Jackson, 2001), mollusks (Foltan and Konvicka, 2008), snakes (Furman et al., 2011), fish (Tiffan et al., 2013), and mammals (Lemen and Freeman, 1985).

Open-source toolkits, such as DeepLabCut (DLC) (Mathis et al., 2018) and DeepPoseKit (DPK) (Graving et al., 2019), utilize deep neural networks (DNNs) for markerless pose estimation directly from video recordings. A workflow based on these toolkits requires two primary steps: training and inference. Training requires annotating key points of interest within the video data, which are then used for training the DNN (Mathis et al., 2018; Graving et al., 2019). The subsequent inference steps when applying the trained neural network to new, unseen video data to predict pose estimates, does not require human involvement, making it an efficient process (Mathis et al., 2018; Graving et al., 2019). The resulting key point tracking data can then be used by tools like BehaviorDEPOT to calculate kinematic and postural statistics and detect behaviors using rule-based heuristics (Gabriel et al., 2022).

To further reduce human labor in the training process, we have developed a method that leverages fluorescent markers for marker-based pose estimation during the data acquisition phase to generate automatically annotated image datasets of rodent paw placement in laboratory setups. Our method relies on a dual-light system of UV and white lights that alternate to illuminate successive frames in a single video stream with two interleaved modalities: a UV and a white light modality. Fluorescent color markers, visible only during UV exposure, distinctly mark the separate paws on UV-exposed frames. We employed a semisupervised cross-modal labeling strategy (Mandal et al., 2020) that used the positional information of UV paw markings to label the white light modality frames. A marker-based pose estimation algorithm filters UV-exposed video frames by paw-specific marker colors to identify and locate the paws. It then deterministically maps these visible marker locations detected on UV-exposed video frames to the paw positions on adjacent white light-exposed frames, taken only 1.4 ms later. This results in a white light-illuminated markerless video with identified paw locations. This automatically labeled video is subsequently used to train a neural network using DLC. During the experimental phase, the trained neural network performs markerless pose estimation directly from video, eliminating the need for markers or UV lights.

We developed our method using a KineWheel–DeepLabCut setup, an open-source experimental setup for markerless paw tracking of head-fixed mice on top of a transparent wheel with an integrated 45° mirror (Warren et al., 2021). For our work we extended this setup with a high-speed camera, dual white and UV lighting, and a controller board for synchronizing frame-by-frame illumination.

## Materials and Methods

**Figure 1.**
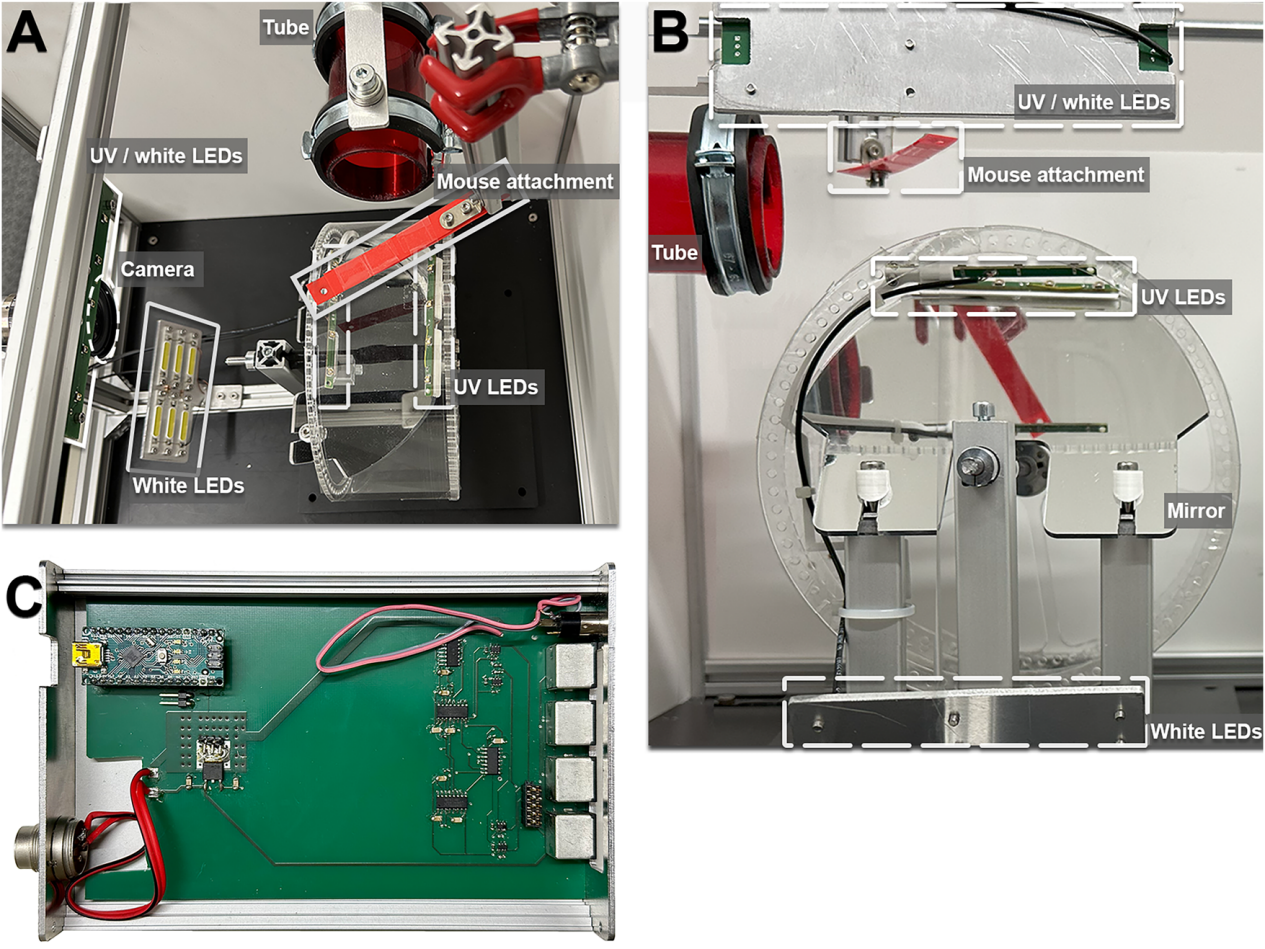
KineWheel experimental setup. ***A***, Top view with the wheel, the mouse attachment above, and the red tube at level with the wheel surface. From within the transparent wheel, two rows of UV lights illuminate the mouse from underneath. Additional lights were placed above and below the camera on the left. ***B***, Camera view of the KineWheel with mirror inside the top half of the wheel. The surrounding white walls contributed to even illumination and blocked stray laboratory light. ***C***, Control circuit with Arduino Nano and connectors for power supply, the four LED modules, and the camera trigger.

The KineWheel (Warren et al., 2021; LABmaker) was placed within a box of 30 cm white foam boards, open to the front (Fig. 1). This box shielded the setup from external light. The matte texture and white color of the boards eliminated reflections and provided a neutral background for video recording with consistent lighting conditions. The top cover was hinged to allow easy access to the wheel when opened. A high-speed color camera (Basler acA720-520uc, up to 720 Hz, global shutter) was mounted at a distance of ∼10 cm in front of the wheel. The camera was tilted down to 7° to capture both the wheel surface in full width and the mirror-mounted inside the KineWheel. We used a 5 mm fixed focal length lens (Computar H0514-MP2) with a large aperture diameter of f/1.4 that allowed exposure times short enough to capture images at a rate of up to 720 Hz. To the left of the wheel, we placed a tube taken from the home cage of the mice that was at eye level but out of reach. The familiar smell of the tube motivated the mice to walk toward it.

**Figure 2.**
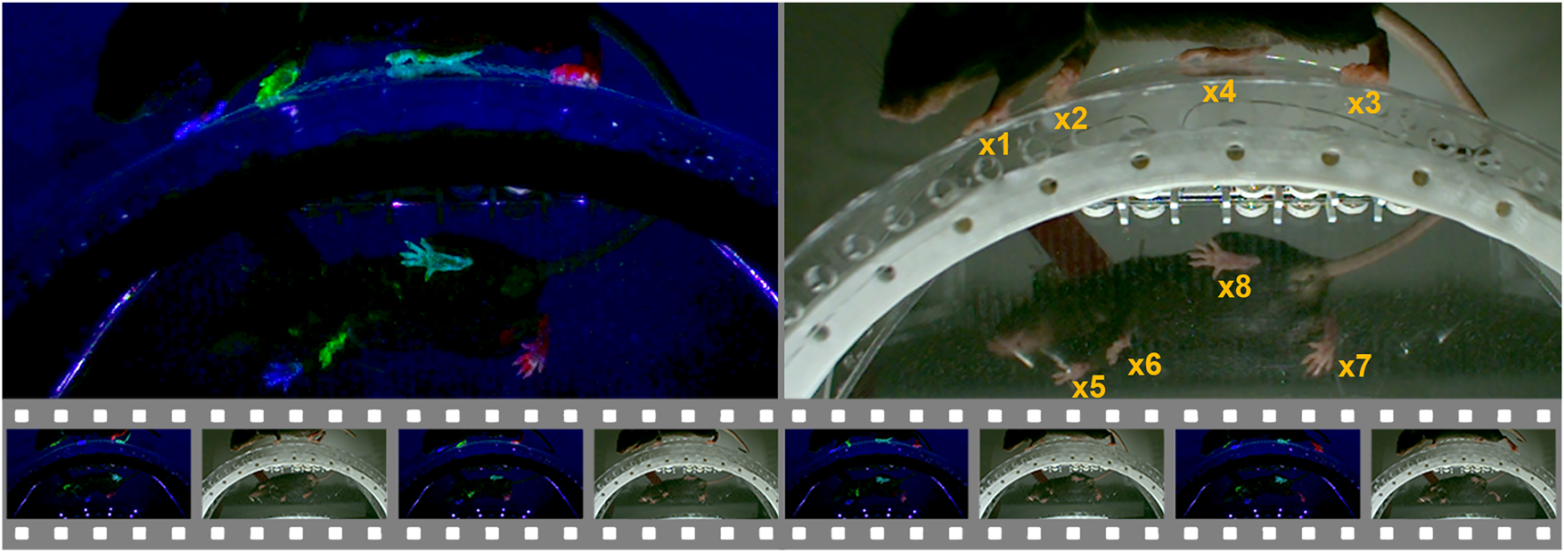
Image sequence illuminated with UV (left) or white light (right). Left, Mouse under UV light (colors enhanced for illustrative purposes). Right, Mouse under white light. Bottom, Consecutive original video sequence of alternating UV and white light illumination.

We provided uniform illumination of the animal through four separately controllable LED light modules. One was mounted inside the wheel to illuminate the underside of the mouse that was visible from the mirror. Two modules, with 10 white light and five UV LEDs, were to the left and the right of the mouse. The fourth white light was mounted on the floor board facing the wheel and tilted upward. Our control board with Arduino Nano (R3) allowed the user to set the operating mode of the system, start and stop video image capture, and adjust the capture rate between 1 and 720 Hz using our control software, KWA-Controller running on a USB connected Windows computer. KWA-Controller is implemented in C++/CLI on .NET framework with Visual Studio 2022. It also controlled the duration of the stroboscopic LED flashes in synchrony with triggering the Basler camera global shutter. The brief flash durations of ∼1 ms froze all movement in the image and effectively increased image sharpness. The two operating modes were inference mode and training mode. In inference mode, only white light LEDs were turned on. This mode was used when the video was to be analyzed by a trained neural network for predicting paw locations. In training mode, the UV and white light LEDs were turned on alternately, creating image series as shown in Figure 2. This mode was used to create automatically annotated datasets that were used to fine-tune a pretrained neural network for detecting the positions of the individual paws.

An odorless, UV-reflective ink (Invisible Pigment Dispersion Concentrate, UV-Elements) was used to mark the paws of a mouse during training mode. The ink emits light in the visible range when illuminated with UV light (365 nm). The ink is water-based, easily washed off with water, and used in products such as body paint for humans. We marked each paw with a different color and selected the four colors to be as different as possible to reduce ambiguity in the color-to-paw filtering and mapping. We used the UV colors green, red, blue (product IDs 337005-06885, 337005- 106 06886, 337005-06887 from Invisible Pigment Dispersion Concentrate, UV-Elements), and turquoise (by mixing blue and green).

We explored different methods of applying the ink. Pipette application, while precise in the amount of ink applied, did not allow for an even distribution and thus resulted in inconsistent markings across paws and mice. Spray application resulted in an even distribution of ink. However, to limit the area of spray application, we used a cover to expose only the paw. This, in turn, resulted in a handling of the animal that we considered to be unnecessarily stressful. Brush application, the method finally used, provided a precise and even distribution of the ink. To ensure consistent ink application, we illuminated a paw with a small UV flashlight during marking.

Marking was done just prior to recording to ensure high color intensity and to avoid contamination of unmarked areas. Each color of ink was applied with a separate brush to maintain color purity. With the mouse held in supine position, marking took ∼1.5 min for all four paws and an additional 30 s to allow the ink to dry. After marking, the mouse was placed on a clean surface to move freely for another minute, which removed excess ink.

**Figure 3.**
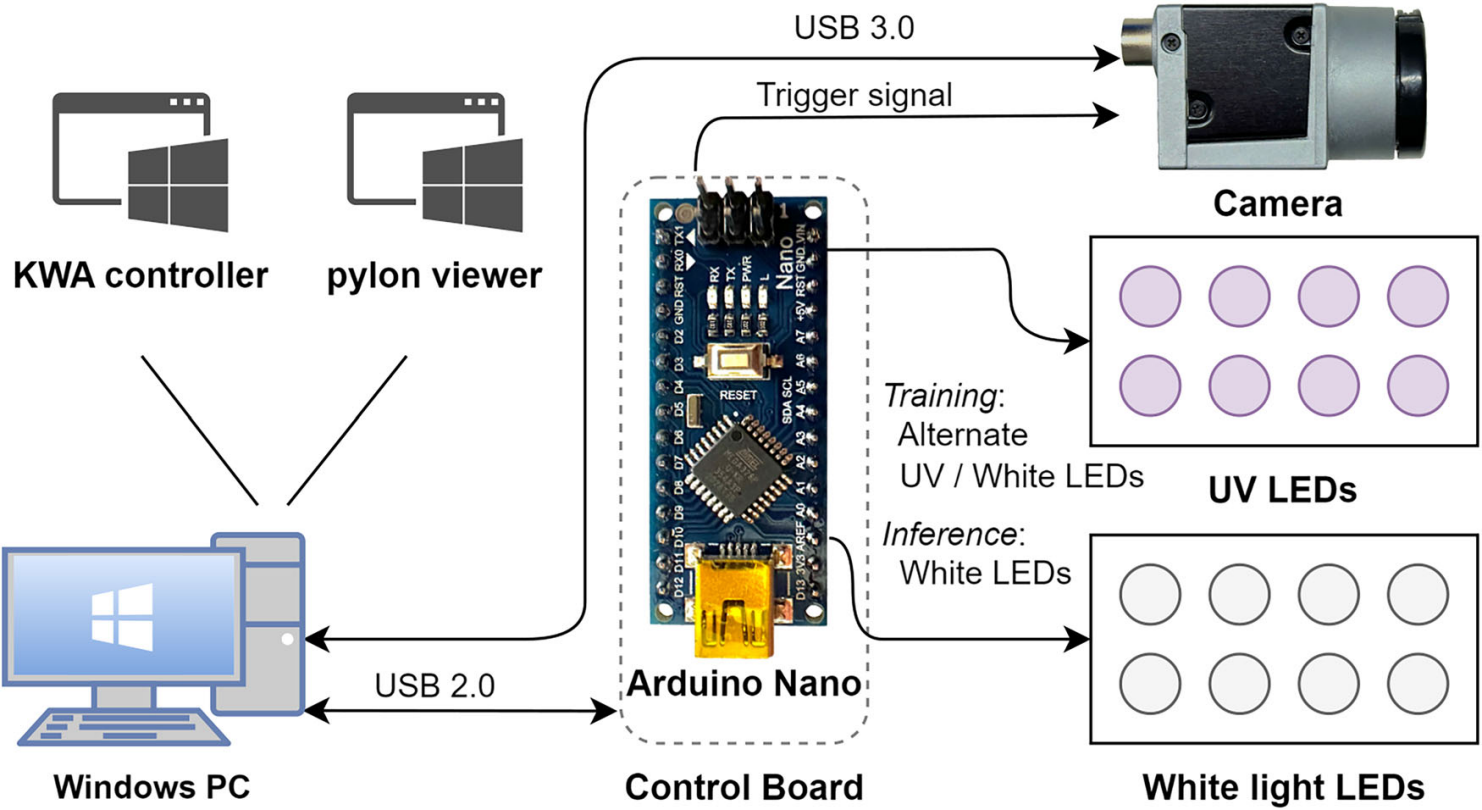
System diagram of main components and connections. The Windows PC runs KWA-Controller interfacing with the Arduino Nano, and pylon Viewer for Basler camera control. Camera connected via USB 3.0 and Arduino via USB 2.0.

We did not head-fix mice for the wheel running training of this study. Instead, the mouse was strapped into a custom-made harness, placed on the wheel, and fixed to an elastic plastic holder (Fig. 1). As the mouse became accustomed to its new environment and started to run toward the tube, the LED lighting was turned on in KWA-Controller, and a video recording was started in pylon Viewer (Fig. 3). Each mouse was recorded for 1–2 min. Because the UV and white light LEDs alternated in synchrony with the camera shutter, the UV markers were visible only on alternating video frames (Fig. 2).

Our custom Python script automatically generated annotations of paw positions in all video frames with regular white light illumination by using the tracking information obtained from the previous UV-illuminated frame. This information was stored in DeepLabCut supported format. Constants in the script defined upper and lower bounds for each marker color and a mapping of marker colors to individual paws. The bounds were specified by pairs of hue, saturation, brightness (HSB) triples and were used to filter markers in UV-illuminated frames (UV frames). To determine the HSB bounds, we selected a small sample of UV frames from the recorded video and loaded them into a Python GUI application that displayed and dynamically updated the filtered versions of the images in response to changes in the user-adjustable HSB bounds. Since the position of the wheel was fixed with respect to the camera, we were able to define binary pixel masks for the wheel surface and mirror view that, when applied to a video frame, preserved only the pixel values in unmasked regions. This allowed us to annotate a total of eight points of interest (four paws, two views) with only four different marker colors.

### Experimental animals

For our recordings we used five female mice (C57BL/6rj), aged 14 months. This work was performed under the supervision of the animal care officer of the Humboldt University of Berlin (Germany) in compliance with EU and state regulations.

### Code accessibility

The code/software described in the article is open source and is freely available on GitHub (https://github.com/WinterLab-Berlin/KineWheelSystem). The code is also available in Extended Data 1. Detailed instructions on how to install and configure the required software and how to record video can be found in the online documentation of KineWheel–DeepLabCut (https://kinewheelsystem.readthedocs.io). The trained neural network weights and labeled/unlabeled sample videos are on Zenodo (https://doi.org/10.5281/zenodo.7586688).

10.1523/ENEURO.0304-23.2024.d1 Extended Data Extended data includes the copy of the GitHub repository. The “Arduino” folder contains the sketch to drive the LEDs and to trigger the camera. This folder contains also the “KWA-Controller” a Windows application to control the Arduino sketch. Inside the “camera” folder are the used camera presets. A very simple Jupyter notebook to run the inference is in the “Inference” folder. The “docs” folder contains the software documentation with instructions and examples on how to use and configure it. The same documentation is also available over the “Read the Docs” webpage. Download Extended Data, ZIP file.

## Results

We recorded video sequences of five mice with a resolution of 720 by 364 pixels at 720 Hz, with video frames alternating between UV and white light exposure. Prior to recording, paws had been marked with four distinct UV ink colors: blue for the left front paw, green for the right front paw, red for the left hindpaw, and turquoise for the right hindpaw. Extracted position of these distinct markings acted as key points for the network to learn and identify. As each video frame captured two nonoverlapping views, specifically a bottom and a side view of a given mouse, a total of eight key points had to be tracked.

For model training and evaluation, a set of 150 white light-exposed images was extracted from the recorded video sequences, with images randomly selected using the *k*-means algorithm provided with the DLC toolkit (Nath et al., 2019), which automated the selection process and ensured high image diversity by selecting a wide variety of animal poses. These images were randomly split into a training and test dataset of 100 and 50 images, respectively. Both training datasets were annotated twice: once manually by a human and once automatically using our script, as described in the Materials and Methods section. The test dataset, serving as the ground truth for evaluation, was annotated once manually. Human annotation was performed by a single person using the labeling GUI in the DLC toolkit and took ∼50 s per image, resulting in a total of 125 min plus an additional 25 min to verify all labels. For consistency, both annotation methods annotated the paws with labels placed near the center of mass of the corresponding, visible paw region.

We used the two labeled datasets to train two separate models and subsequently compared their performance to evaluate the effectiveness of our proposed method. The training process for both models was performed using the DeepLabCut framework with identical training parameters. Both models were trained for 30,000 iterations with snapshots taken every 2,500 iterations. We evaluated the last five snapshots on the same test dataset and selected the best performing snapshot of each model for comparison.

### Model performance

To compare the performance of the models trained on the automatically and manually annotated data, an independent two-sample *t* test was performed. The model trained on the automatically annotated dataset achieved a mean Euclidean distance of 2.6 pixels (SEM = 0.23), while the model trained on the manually annotated dataset achieved a mean Euclidean distance of 2.7 pixels (SEM = 0.17). The *t* test revealed no significant difference between the two models (*t*_(48)_ = 0.35; *p* = 0.73). These results suggest that the model trained on the automatically annotated data performed as well as the model trained on the manually annotated data.

**Figure 4.**
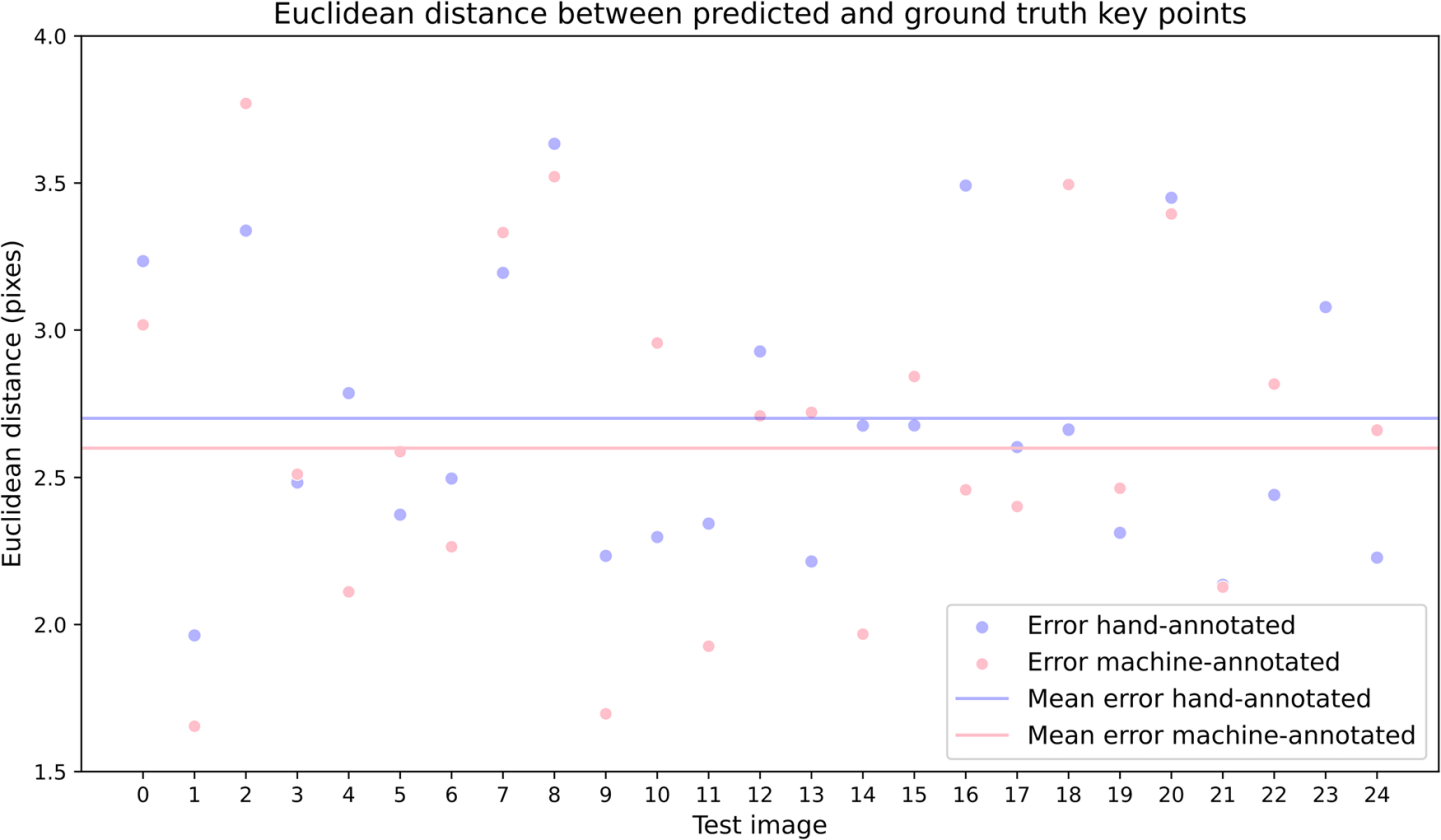
Euclidean distances between predicted key points from a neural network model trained on machine-annotated images (pink circles) and human-annotated images (blue circles), compared with the ground truth key points. Each point represents 1 of 25 randomly selected test images, with the *x*-axis indicating the test image number (0–24) and the *y*-axis representing the Euclidean distance in pixels. Two horizontal lines represent the models' mean Euclidean distance across all test images.

Figure 4 shows the Euclidean distance between the predicted key points from the two neural network models and the ground truth key points for 25 of the 50 test images. The data points are scattered over a range of ∼1.5–4 pixels on the *y*-axis, which represents the Euclidean distance in pixels. The standard deviation values of 0.68 pixels for the algorithm-annotated model and 0.47 pixels for the human-annotated model are essentially negligible, as one pixel in the mouse paw region represents <1 mm^2^. The subpixel standard deviations suggest that both models perform remarkably well, with their predictions being accurate and consistent with the ground truth key point locations across the test images.

### Efficiency

The use of automatic annotation significantly reduced the time and effort required to create the training dataset. In contrast to the 150 min required for the manual annotation, the automatic annotation process in addition to the initial 3 min painting procedure took only a few minutes, followed by a brief manual verification of the generated annotations.

## Discussion

We present a novel method for automated rodent pose tracking using invisible fluorescent markers, which has the potential to significantly improve the efficiency of data annotation and reduce the need for human labor in the training process of deep neural networks for markerless pose estimation. The results obtained from our experiments demonstrate that our model trained on automatically annotated data performed comparably with the model trained on human-annotated data, suggesting that our method is a viable alternative to traditional manual annotation.

Nevertheless, marker-based pose estimation has limitations that must be considered. One of the main limitations is that placing markers on animals can be invasive and may interfere with their natural movement or behavior (Dennis et al., 2008; Das et al., 2023). However, with our approach, fluorescent markers are only used during the training data acquisition phase to enable automatic annotation of paw positions. Markers are not present during the actual experimental phase when the trained neural network is used for markerless paw tracking. Therefore, our use of markers on rodent paws does not affect behavior during the experiments. Additionally, markers can wear off, degrade, or become obscured in long-duration studies or under certain conditions, such as when the animal licks or rubs its paws (Klabukov et al., 2023). In our case, fluorescent markers only needed to last for the duration of a single recording session. The water-based ink dried quickly and adhered well to animal paws, remaining visible under UV illumination throughout the recording session. Thus, long-term durability was not a concern. Setting up marker-based systems can be a time-consuming process, as each marker needs to be placed precisely on the subject (Das et al., 2023). In contrast, applying fluorescent markers to rodent paws requires some manual effort but this is one-time only for the training session. The automatic annotation procedure then allows for the generation of large, labeled datasets from a video captured with the alternating UV/white light setup. This training data is generated much more efficiently than by manually clicking key points in hundreds or thousands of frames. Our method relies on accurately identifying and locating key points of interest based on the distinct colors of the fluorescent markers. If the number of required markers exceeds the number of available, well-separable colors, the quality of the automatically generated annotations may suffer. However, for only four paws it was easy for us to identify separable colors that the color filtering algorithm could reliably distinguish. Therefore, limited scalability was not a constraint in our application.

Our automated annotation approach offers significant benefits in terms of efficiency, reduction of manual labor, and minimization of human bias, while achieving annotation quality comparable with manual labeling. This increased efficiency could potentially enable researchers to analyze larger datasets and generate more robust models for pose estimation. Furthermore, by illuminating video images stroboscopically with brief flash durations, we froze all movement in a frame which effectively increased image sharpness.

The development of accurate, automated, and efficient methods for rodent pose tracking is crucial for advancing this method in neuroscience research aimed at understanding the complex relationships between neural circuits, behavior, and neurological disorders. Our proposed method offers a promising alternative to manual annotation. Future work to further validate the effectiveness of our method in an increasing variety of experimental settings and across different species could take this method to transparent treadmills, transparent bottom arenas, or runways for gait analysis.

## Synthesis

Reviewing Editor: Christine Portfors, Washington State University Decisions are customarily a result of the Reviewing Editor and the peer reviewers coming together and discussing their recommendations until a consensus is reached. When revisions are invited, a fact-based synthesis statement explaining their decision and outlining what is needed to prepare a revision will be listed below. The following reviewer(s) agreed to reveal their identity: NONE.

Synthesis:

The reviewers agree that providing a simple method for automating rodent pose tracking with UV light is an important methodological advance. This method could lead to advances in behavior and circuitry. However, the reviewers agree that the results section would benefit from additional control experiments, data analysis, statistical analysis and data figures. The manuscript also suffers throughout from a lack of references to relevant literature.

Reviewers' comments:

Major comments:

Novelty: This trick is almost classic and the authors should make an effort to find a few references. Firstly, with UV light they basically perform marker-based pose estimation. Secondly, there are several papers on cross-modal labeling. Importantly, I do not think that the lack of novelty impacts publication in eNeuro, as they provide hardware, basic code and some validation. However, they should look for the relevant references and as I will discuss below, they should also improve the quality of the result section.

Generalizability: Naturally, I do think that this is a useful trick. However, for readers it should be demonstrated that you can do the following: Collect paired data from a few sessions/mice, and then have a DLC model for which the paired data is no longer necessary (and the UV channel) to analyze poses based on RGB imaging.

Limitations: As the method relies on markers, all relevant limitations for marker-based pose estimation applies. Those should be discussed; you can find them in relevant work.

Stats and Quantification: Line 183+ you only compare mean values without any tests. Yet, you do have 50 paired test images. So why don't you report SEM and also carry out some statistical test. Furthermore, the quality of the marker-based and human annotations should be better analyzed and compared (on an image-by-image basis). You may take some inspiration of the figures in DeepLabCut or other relevant publications. The learning curve of the loss is not particularly important as a main figure. Consider showing the evaluation vs. training as this has more meaning; also include the generalization results in this figure, i.e., how well do the different models generalize to novel videos.

Another issue with the paper is some lack of behavior controls. Ink is being painted on the rat paws and, while I very much appreciate the correctness in their choosing the least stressful way to do this, they report giving very-little to no acclimation time before running the rats on the wheel assay. Further, they do not provide individual data sets for each of the 5 rats. Does the ink affect the rats ability to run on the wheel? Would the total loss graphs of machine annotated+ink and machine annotated-no ink look different? They are missing some proof-of-concept application controls from which the story could really benefit.

Additionally, the paper does not reference, cite, or address the work reported in {Gabriel et al.2022 BehaviorDEPOT is a simple, flexible tool for automated behavioral detection based on markerless pose tracking. eLife https://doi.org/10.7554/eLife.74314}. I think this is an oversight, as Gabriel et al. asserts using markerless pose tracking and this paper uses marker post tracking.

Minor comments:

- Citations: In general, this article has barely any citations; the discussion has none etc. This should also be rectified. E.g., relevant reviews on the topics should be included so that readers can find those references. Also, it appears that no work past 2019 is cited, but that's the duration of a Phd thesis past, and about 8 Master thesis cycles!

- For the mouse pose annotation dataset, 64 videos were sampled from data over many years, what is the relationship to the other dataset, if any?

- Line 32, the intro is misleading as the problem and the solution both cite Mathis/Pereira. Unclear why Wiltschko is in this list. Just cite some reviews on pre deep learning methods, e.g., Mathis et. al 2020 Neuron and related refs.

- Fig 1: perhaps move the letters out of the images to increase contrast.

- Line 163: Nath et al. Nat Protocols should be cited as this uses DLC 2.+

## Author Response

Response to the Reviewer's comments We thank you for reviewing our manuscript, "KineWheel-DeepLabCut automated paw annotation using alternating stroboscopic UV and white light,". We have carefully addressed your comments and made the necessary revisions to the manuscript. Please find our point-by-point responses below.

Major Comments Novelty The introduction section has been enhanced by the addition of two paragraphs that provide an overview of the history of pose estimation methods and fluorescent markers. These paragraphs are accompanied by a comprehensive list of references.

Generalizability We have elaborated on what our method entails and at which step in the process -- data acquisition and neural network training &inference -- it is applied. The second to last paragraph of the introduction section highlights the difference between the data acquisition and inference phases, where the latter phase does not require a UV modality or markings on the rodent's paws.

Limitations We have now added two paragraphs to the Discussion section, explaining the limitations of marker-based pose estimation and citing relevant work.

Effect of ink on mice running on wheel We have added a sentence to the Methods section to address the effect of the ink on the mice's ability to run on the wheel. We point out that we never used the marker-based paw detection directly in any experiments on kinematics, but only during the data acquisition phase to generate large quantities of consistently and deterministically annotated rodent paws for training the neural network. The ink dries quickly and does not affect the ability of the mice to run on the wheel.

Reference BehaviorDepot We have added the important paper by Gabriel et al. (2022) on BehaviorDepot to the introduction section, along with references to DeepLabCut and DeepPoseKit.

2 Statistics and quantification We have improved the Results section to include a two-sample t-test and replaced the old Figure 4 with a scatterplot of the model's errors on paired test images.

Minor comments Add citations to the introduction and discussion section We have added further relevant references to the introduction and discussion section, covering both historical and current research on the use of fluorescent markers and limitations of marker-based pose estimation.

Reviews on pre-DeepLearning We have updated the references in the first paragraph of the introduction section, comparing traditional (pre-DeepLearning) pose estimation methods to automated methods.

Mouse pose annotation dataset (64 videos) We apologize for the confusion regarding this comment. Could you please clarify which specific data in the manuscript you are referring to? We would be happy to address this point once we have a better understanding of the context.

Improve Figure 1 We have followed this good advice and moved the letters of the images in Figure 1 to avoid interfering with the content, making them clearly visible.

DLC 2.+ reference We have cited the Nath et al. Nature Protocols paper since we are using DeepLabCut version 2 and later in our work.

We appreciate the thorough review and the opportunity to improve our manuscript. We believe that the revisions have addressed the reviewers' concerns and enhanced the quality of our work. Please let us know if any further clarification or modifications are needed.

Thank you for your consideration.
