# Supplementary figures and images for: KineWheel–DeepLabCut Automated Paw Annotation Using Alternating Stroboscopic UV and White Light Illumination
Source: eNeuro. 2024 Aug 23;11(8):ENEURO.0304-23.2024. doi: 10.1523/ENEURO.0304-23.2024 (PMC11363514; doi:10.1523/ENEURO.0304-23.2024)

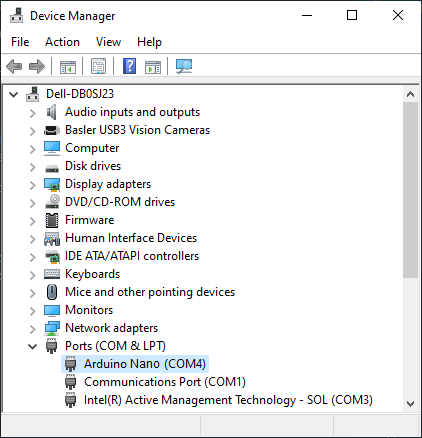

Supplement: Extended Data — includes the copy of the GitHub repository. The “Arduino” folder contains the sketch to drive the LEDs and to trigger the camera. This folder contains also the “KWA-Controller” a Windows application to control the Arduino sketch. Inside the “camera” folder are the used camera presets. A very simple Jupyter notebook to run the inference is in the “Inference” folder. The “docs” folder contains the software documentation with instructions and examples on how to use and configure it. The same documentation is also available over the “Read the Docs” webpage. Download Extended Data, ZIP file. [file eneuro-11-ENEURO.0304-23.2024-s002.zip › docs/source/media/Arduino-Nano-In-Device-Manager.png]

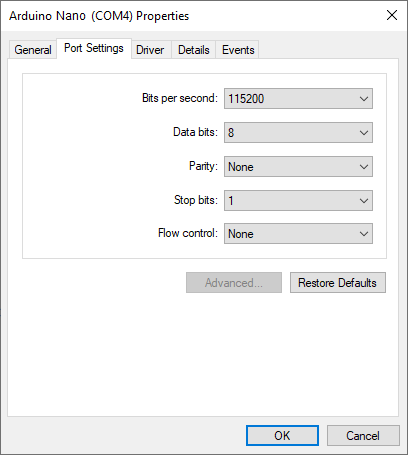

Supplement: Extended Data — includes the copy of the GitHub repository. The “Arduino” folder contains the sketch to drive the LEDs and to trigger the camera. This folder contains also the “KWA-Controller” a Windows application to control the Arduino sketch. Inside the “camera” folder are the used camera presets. A very simple Jupyter notebook to run the inference is in the “Inference” folder. The “docs” folder contains the software documentation with instructions and examples on how to use and configure it. The same documentation is also available over the “Read the Docs” webpage. Download Extended Data, ZIP file. [file eneuro-11-ENEURO.0304-23.2024-s002.zip › docs/source/media/Arduino-Nano-Port-Settings-Device-Manager.png]

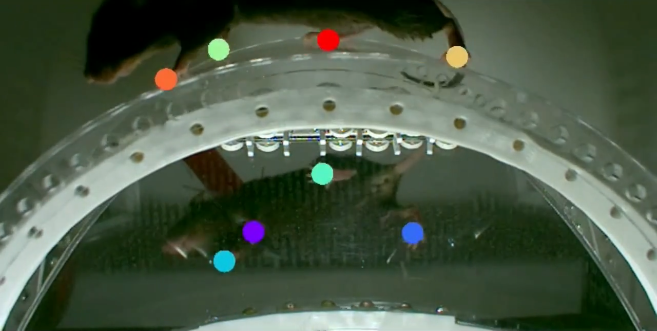

Supplement: Extended Data — includes the copy of the GitHub repository. The “Arduino” folder contains the sketch to drive the LEDs and to trigger the camera. This folder contains also the “KWA-Controller” a Windows application to control the Arduino sketch. Inside the “camera” folder are the used camera presets. A very simple Jupyter notebook to run the inference is in the “Inference” folder. The “docs” folder contains the software documentation with instructions and examples on how to use and configure it. The same documentation is also available over the “Read the Docs” webpage. Download Extended Data, ZIP file. [file eneuro-11-ENEURO.0304-23.2024-s002.zip › docs/source/media/DNN-Annotated-Video-Frame.png]

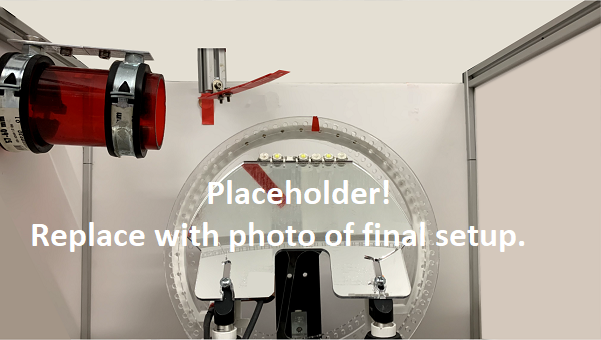

Supplement: Extended Data — includes the copy of the GitHub repository. The “Arduino” folder contains the sketch to drive the LEDs and to trigger the camera. This folder contains also the “KWA-Controller” a Windows application to control the Arduino sketch. Inside the “camera” folder are the used camera presets. A very simple Jupyter notebook to run the inference is in the “Inference” folder. The “docs” folder contains the software documentation with instructions and examples on how to use and configure it. The same documentation is also available over the “Read the Docs” webpage. Download Extended Data, ZIP file. [file eneuro-11-ENEURO.0304-23.2024-s002.zip › docs/source/media/Experimental-Setup-Camera-View.png]

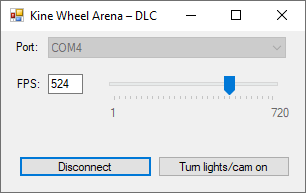

Supplement: Extended Data — includes the copy of the GitHub repository. The “Arduino” folder contains the sketch to drive the LEDs and to trigger the camera. This folder contains also the “KWA-Controller” a Windows application to control the Arduino sketch. Inside the “camera” folder are the used camera presets. A very simple Jupyter notebook to run the inference is in the “Inference” folder. The “docs” folder contains the software documentation with instructions and examples on how to use and configure it. The same documentation is also available over the “Read the Docs” webpage. Download Extended Data, ZIP file. [file eneuro-11-ENEURO.0304-23.2024-s002.zip › docs/source/media/KWA-Controller-App-1.png]

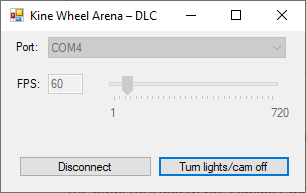

Supplement: Extended Data — includes the copy of the GitHub repository. The “Arduino” folder contains the sketch to drive the LEDs and to trigger the camera. This folder contains also the “KWA-Controller” a Windows application to control the Arduino sketch. Inside the “camera” folder are the used camera presets. A very simple Jupyter notebook to run the inference is in the “Inference” folder. The “docs” folder contains the software documentation with instructions and examples on how to use and configure it. The same documentation is also available over the “Read the Docs” webpage. Download Extended Data, ZIP file. [file eneuro-11-ENEURO.0304-23.2024-s002.zip › docs/source/media/KWA-Controller-App-On-Port-COM4-At-60-FPS.png]

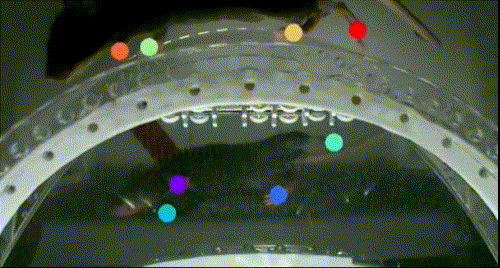

Supplement: Extended Data — includes the copy of the GitHub repository. The “Arduino” folder contains the sketch to drive the LEDs and to trigger the camera. This folder contains also the “KWA-Controller” a Windows application to control the Arduino sketch. Inside the “camera” folder are the used camera presets. A very simple Jupyter notebook to run the inference is in the “Inference” folder. The “docs” folder contains the software documentation with instructions and examples on how to use and configure it. The same documentation is also available over the “Read the Docs” webpage. Download Extended Data, ZIP file. [file eneuro-11-ENEURO.0304-23.2024-s002.zip › docs/source/media/Labeled-Video-Sample-LowRes-12FPS.gif]

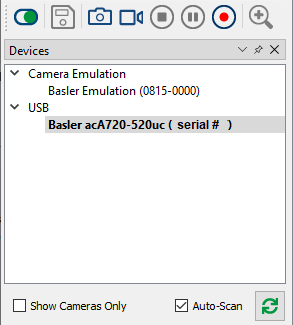

Supplement: Extended Data — includes the copy of the GitHub repository. The “Arduino” folder contains the sketch to drive the LEDs and to trigger the camera. This folder contains also the “KWA-Controller” a Windows application to control the Arduino sketch. Inside the “camera” folder are the used camera presets. A very simple Jupyter notebook to run the inference is in the “Inference” folder. The “docs” folder contains the software documentation with instructions and examples on how to use and configure it. The same documentation is also available over the “Read the Docs” webpage. Download Extended Data, ZIP file. [file eneuro-11-ENEURO.0304-23.2024-s002.zip › docs/source/media/pylon-Viewer-Open-Device.png]

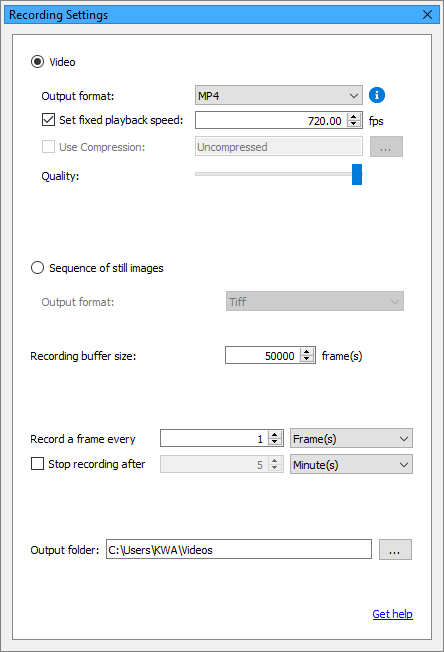

Supplement: Extended Data — includes the copy of the GitHub repository. The “Arduino” folder contains the sketch to drive the LEDs and to trigger the camera. This folder contains also the “KWA-Controller” a Windows application to control the Arduino sketch. Inside the “camera” folder are the used camera presets. A very simple Jupyter notebook to run the inference is in the “Inference” folder. The “docs” folder contains the software documentation with instructions and examples on how to use and configure it. The same documentation is also available over the “Read the Docs” webpage. Download Extended Data, ZIP file. [file eneuro-11-ENEURO.0304-23.2024-s002.zip › docs/source/media/pylon-Viewer-Recording-Settings.png]

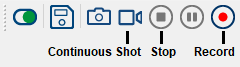

Supplement: Extended Data — includes the copy of the GitHub repository. The “Arduino” folder contains the sketch to drive the LEDs and to trigger the camera. This folder contains also the “KWA-Controller” a Windows application to control the Arduino sketch. Inside the “camera” folder are the used camera presets. A very simple Jupyter notebook to run the inference is in the “Inference” folder. The “docs” folder contains the software documentation with instructions and examples on how to use and configure it. The same documentation is also available over the “Read the Docs” webpage. Download Extended Data, ZIP file. [file eneuro-11-ENEURO.0304-23.2024-s002.zip › docs/source/media/pylon-Viewer-Toolbar.png]
